# Oxidants, antioxidants, and the clinical course of COVID-19 disease: a prospective observational study

**DOI:** 10.1186/s12985-026-03124-2

**Published:** 2026-03-23

**Authors:** Werner Dammermann, Wencke Schürmann, Till Bornscheuer, Stefan Lüth, Dominique Petrus, Sandra Schwarzlose-Schwarck, Florian Hentschel

**Affiliations:** 1 Department of Gastroenterology, Diabetology and Hepatology, University Hospital Brandenburg, Brandenburg Medical School Theodor Fontane, Brandenburg an Der Havel, Germany; 2 Department of Cardiology, University Hospital Brandenburg, Brandenburg Medical School Theodor Fontane, Brandenburg an Der Havel, Germany; 3 Center for Translational Medicine, Brandenburg Medical School Theodor Fontane, Germany, Brandenburg an Der Havel, Germany; 4 Department of Oncology, University Hospital Brandenburg, Brandenburg Medical School Theodor Fontane, Brandenburg an Der Havel, Germany; 5 MSB Medical School Berlin, Berlin, Germany; 6 Helios Klinikum Bad Saarow, Pieskower Straße 33, 15526 Bad Saarow, Germany

**Keywords:** Xanthine oxidase, Superoxide dismutase, Catalase, Ischemia modified albumin, Reactive oxygen species, Oxidative stress, COVID-19

## Abstract

**Background:**

Microvascular damage by oxidative stress is central in the pathogenesis of generalized COVID-19 disease. Hence, a disbalance of endothelial-derived oxidative and anti-oxidative factors in COVID-19 patients can be expected, and the extent of this disbalance might correlate with disease severity.

**Methods:**

We measured xanthine oxidase (XO), superoxide dismutase (SOD), catalase (CAT), and ischemia modified albumin (IMA) in serum samples of 166 COVID-19 patients and 238 controls. We then cathegorized the COVID-19 group further into mild, moderate, severe and lethal courses and tested these for correlation with each parameter alone, and with multi-parametric logistic regression analysis.

**Results:**

Compared to controls, XO was significantly lower in COVID-19 patients, SOD and CAT were significantly higher. Difference in IMA was insignificant. In the single parameter analysis, only CAT concentration was significantly correlated to disease severity. In the logistic regression analysis, XO and SOD were negatively correlated with disease severity.

**Conclusions:**

Oxidative stress in COVID-19 does derive from other sources than endothelial XO. The rise in protective enzymes like SOD and CAT may be the result of enzyme induction. Since the correlation of CAT with disease severity was highest, we propose this parameter as a possible predictor for a severe clinical course.

## Introduction

Not long after the first description of COVID-19 disease, it became clear that it does not solely affect the lung [1, 2]. Since the virus particles enter cells via the ACE2 receptor, all cells expressing this receptor can be infected [3]. Correspondingly, COVID-19 has been found in the gut, kidney, heart, skin, and notably in the vascular endothelium [4–6].

Additionally, it was discovered that the damage caused by COVID-19 is not necessarily limited to the infected cells alone. In contrary, the excessive inflammatory response that results in the proverbial cytokine storm can damage all cells of the body, making COVID-19 a multi-systemic disease [7, 8].

One ubiquitous cell type that is a contributor as well as a target for this inflammatory response is, again, the vascular endothelium [9, 10]. Here, the cytokine release will lead to the formation of reactive oxygen species (ROS), like superoxide anion (O2^•−^) or hydrogen peroxide (H_2_O_2_)_._ These ROS then harm the microvasculature either via direct oxidation of proteins or lipids, or by decreasing the effect of nitric oxide [11–16]. Vice versa, ROS can also increase the activity of pro-inflammatory cytokines via the recruitment of neutrophils and monocytes [17, 18]. The result is a vicious circle that will eventually lead to a complete breakdown of the microcirculation [19].

In healthy individuals, oxidative stress is normally mitigated by various protective factors, or antioxidants, that deactivate the ROS before they damage other structures [20]. In COVID-19, as in other inflammatory diseases, this homeostasis is thought to be out of balance [21, 22]. So in this context, it is not surprising that COVID-19 has been called “a hyper-inflammatory disease”, or even “a vascular disease” [23–25].

Clinically, the course of the disease is the more severe, the more generalized the inflammation is and the more the microcirculation is compromised [26–29]. Correspondingly, certain inflammatory markers that can be measured at an early stage of the infection have been shown to predict its further clinical course [30, 31].

Against this background, we suspect that an imbalance of endothelium-related oxidants and antioxidants can also be detected in real-life COVID-19 patients, and we further hypothesize that the extent of oxidative stress might be a marker of the severity of the disease [32].

An ideal method to quantify this extent of oxidative stress would be to measure ROS directly in the vascular endothelium [33]. Unfortunately, these molecules are difficult to measure in-vivo because they spontaneously react with everything in their vicinity and often vanish as fast as they are produced [34]. So while it is not entirely impossible to detect ROS in some well-defined clinical settings [35, 36], it is usually reserved for cell cultures or animal models [33, 37, 38].

**Fig. 1:**
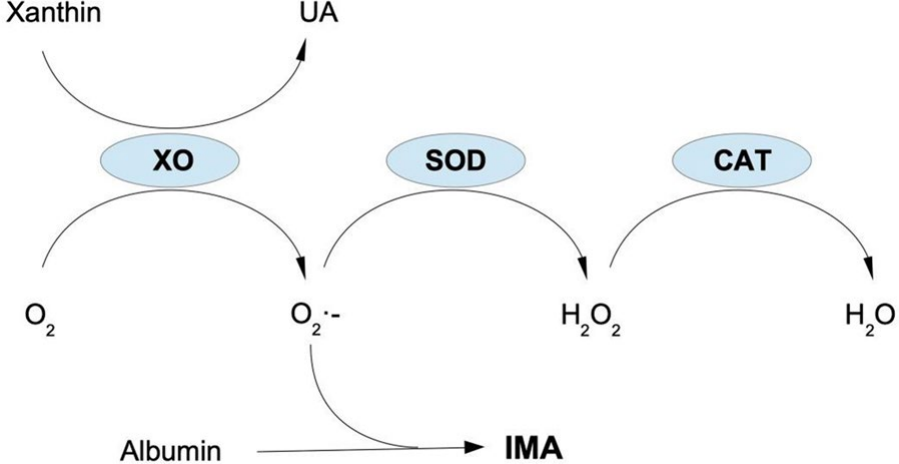
Generation and decomposition of ROS. UA = Uric acid, XO = xanthine oxidase, SOD = superoxide dismuthase, CAT = catalase, IMA = ischemia modified albumin

Therefore, in this prospective clinical observational study, we measured well-established *indirect* markers of oxidative endothelial stress as well as protective factors against oxidative endothelial stress in 238 healthy controls and 166 COVID-19 patients, and compared the results with the clinical severity of the disease. These markers were (Fig. 1):

- Xanthine oxidase (XO). XO is abundant in the capillary endothelium and, to a lesser extent, in the alveolar epithelium. Here, it is not just involved in the production of uric acid (UA), but just as much in the generation of the highly reactive superoxide anion O2^•−^ [39–41].
- Superoxide dismutase (SOD). This enzyme can be found extracellularly and in various cell types [42–44]. Its main function is to transform O2^•−^ into the less reactive H_2_O_2_.
- Catalase (CAT). CAT is a ubiquitous enzyme that decomposes H_2_O_2_ into water and oxygen [45].
- Ischemia Modified Albumin (IMA). The properties of Albumin change under oxidative stress, like in acidosis or ischemia. Albumin altered in this way can be detected by ELISA in vivo, and is considered a marker for past oxidative stress [46].

## Material and methods

### Recruitment of study participants

Between March 30, 2020, and May 21, 2021, serum samples were collected from adult patients who tested positive for SARS-CoV-2 by PCR and were hospitalized at the University Hospital Brandenburg an der Havel.

Inclusion criteria were:

- Age more than 18 years
- Symptomatic disease, hospitalized
- Positive SARS-CoV-2 PCR test
- Written informed consent.

Exclusion criteria were:

- Nosocomial SARS-CoV-2 infection
- Medication with allopurinol, febuxostat, and/or albumin
- Known mutations or enzyme defects of XO, SOD, or CAT.

In the COVID-19 group, patients were further categorized by the clinical severeness of their disease using a simplified ordinal scale based on the 2020 WHO Clinical Progression Scale (CPS) [47, 48]:

- Light: Normal ward, no oxygen support, recovered (corresponding to a CPS of 4)
- Moderate: Normal ward, oxygen support, recovered (corresponding to a CPS of 5 and 6)
- Severe: Intensive care unit, mechanical ventilation, recovered (corresponding to a CPS of 7 to 9) (Among these, eleven were transferred from the general ward, and ten were directly admitted to the ICU.)
- Lethal: Intensive care unit, mechanical ventilation, died (corresponding to a CPS of 10).

A control group was established, consisting of healthy volunteers (employees of the University Hospital Brandenburg an der Havel) who had a negative SARS-CoV-2 test at the time of blood collection. A total of 404 participants were included in the study (n = 404). The patient cohort comprised 166 COVID-19 patients (n = 166), referred to as the COVID-19 group. The control group included 238 individuals (n = 238).

### Determination of XO activity using the Amplex® Red Xanthine/Xanthine Oxidase assay kit

The Amplex® Red Xanthine/Xanthine Oxidase Assay-Kit was purchased from Thermo Fisher, USA (# A22182). All reagents from the kit were brought to room temperature (15–25 °C) before the assay. Patient and control sera, previously stored at -20 °C or -80 °C, were thawed at 4 °C. Test strips were inserted into the provided ELISA plate frame, and a protocol sheet was prepared. The first and second rows were designated for the creation of a standard curve (positive and negative controls) and quality assurance, while the remaining rows were used for sample analysis. Amplex Red reagent was reconstituted with 100 µl of dimethyl sulfoxide (DMSO), protected from light, and stored at room temperature until use. Four milliliters of 5 × reaction buffer were diluted with 16 ml of deionized water (ddH_2_O) to a 1:5 ratio. Horseradish peroxidase (HRP) was reconstituted with 200 µl of 1 × reaction buffer, and xanthine oxidase (XO) was mixed with 100 µl of ddH_2_O. A standard curve was prepared by diluting 10 µl of XO standard with 990 µl of 1 × reaction buffer to create a 1:100 pre-dilution. Then, 40 µl of this pre-diluted XO standard were mixed with 360 µl of 1 × reaction buffer to create Standard 1 (10 mU/ml). Further serial dilutions were performed, generating Standards 2 (5 mU/ml), 3 (2.5 mU/ml), 4 (1.25 mU/ml), 5 (0.625 mU/ml), and 6 (0.3125 mU/ml). Two negative controls containing 200 µl of 1 × reaction buffer were included for quality assurance. Serum samples were diluted 1:5, resulting in a dilution of 24 µl of sample with 96 µl of 1 × reaction buffer. Fifty microliters of each standard and sample were pipetted into the wells of the microtiter plate, mixed thoroughly, and incubated in duplicates. The Amplex Red working solution was prepared by mixing 50 µl of Amplex Red reagent, 20 µl of HRP solution, 50 µl of xanthine solution, and 4.88 ml of 1 × reaction buffer. Fifty microliters of this solution were added to each well. The microtiter plate was covered with adhesive film to prevent evaporation and contamination, and incubated at 37 °C for 30 min, protected from light. During the assay, XO catalyzed the oxidation of purine bases, xanthine or hypoxanthine, to uric acid and superoxide. Superoxide spontaneously decomposed into H_2_O_2_, which, in the presence of HRP, reacted stoichiometrically with Amplex Red reagent to produce the red-fluorescent oxidation product resorufin. The absorbance was measured at 560 nm within 30 min using an ELISA photometer, and the mean values were calculated from replicate readings.

### Determination of SOD activity using the superoxide dismutase (SOD) colorimetric activity kit

The Superoxide Dismutase (SOD) Colorimetric Activity Kit was purchased from Thermo Fisher, USA (# EIASODC). All reagents from the kit were brought to room temperature (15–25 °C) before the assay. Patient and control sera, previously stored at -20 °C or -80 °C, were thawed at 4 °C. Test strips were inserted into the provided ELISA plate frame, and a protocol sheet was prepared. The first and second rows were designated for the creation of a standard curve (positive and negative controls) and quality assurance, while the remaining rows were used for sample analysis. The standard curve was first prepared by adding 75 µl of assay buffer to each matrix tube. The SOD standard was diluted with 250 µl of assay buffer, vortexed, and incubated at room temperature for 5 min before use. Next, 75 µl of the reconstituted SOD standard was pipetted into Standard 1. From Standard 1, 75 µl of solution was transferred to Standard 2, and this step was repeated for each successive tube up to Standard 6. Standards 7 and 8 were used as blanks. The SOD standard curve comprised 2 U/ml, 1 U/ml, 0.5 U/ml, 0.25 U/ml, 0.125 U/ml and 0.625 U/ml, respectively.

For the sample preparation, 80 µl of assay buffer was added to each sample well, followed by the addition of 20 µl of sample, resulting in a 1:5 dilution. The mixture was thoroughly resuspended in the matrix tubes. For incubation, 10 µl of each standard or sample was pipetted into the wells of the ELISA plate, and duplicate measurements were performed. In addition, 50 µl of substrate solution (prepared by diluting 500 µl of 10 × substrate solution with 4.5 ml substrate diluent to achieve a 1:10 dilution) was added to each well. To avoid interference from highly colored samples, background absorption was measured at 450 nm using an ELISA photometer before proceeding with the test. These background values were subtracted from the final sample readings. In the second incubation step, 25 µl of xanthine oxidase (XO) solution was added to each well containing standards and samples. The XO solution was prepared by diluting 100 µl of 25 × XO solution with 2.4 ml of XO buffer, achieving a 1:25 dilution. The entire plate was covered with adhesive film to prevent evaporation and contamination, and incubated on a shaker at 200 rpm for 20 min at room temperature. During this incubation, XO generated superoxide in the presence of oxygen, which converted the colorless substrate into a yellow-colored product. At the end of the incubation period, absorbance was measured immediately at 450 nm. The mean values were calculated from all replicates.

### Determination of CAT activity using the catalase (CAT) colorimetric activity kit

The Catalase (CAT) Colorimetric Activity Kit was purchased from Thermo Fisher, USA (# EIACATC). All reagents from the kit were brought to room temperature (15–25 °C) before the assay. Patient and control sera, previously stored at -20 °C or -80 °C, were thawed at 4 °C. Test strips were inserted into the provided ELISA plate frame, and a protocol sheet was prepared. The first and second rows were designated for the creation of a standard curve (positive and negative controls) and quality assurance, while the remaining rows were used for sample analysis. A 1:5 dilution of 14 ml of assay buffer (5x) was prepared with 56 ml of deionized water (ddH_2_O). Additionally, a 1:50 dilution of the HRP solution was prepared by mixing 50 µl of HRP solution (50x) with 2.45 ml of 1 × assay buffer. The CAT standard curve comprised 5 U/ml, 2.5 U/ml, 1.25 U/ml, 0.625 U/ml, 0.313 U/ml and 0.156 U/ml, respectively. Serum samples were diluted 1:25 by adding 96 µl of 1 × assay buffer and 4 µl of serum sample to each matrix tube, followed by thorough resuspension. For incubation, 25 µl of either standard, sample, or 1 × assay buffer were pipetted into each well in duplicate. Following the protocol, 25 µl of H2O2 was added to each well. The microtiter plate was covered with adhesive film to prevent evaporation and contamination and incubated at room temperature on a shaker at 200 rpm for 30 min. After incubation, 25 µl of the colorimetric detection reagent (substrate) was added to each well, followed by 25 µl of the pre-diluted HRP solution. The plate was again sealed with adhesive film and incubated for 15 min at room temperature on the shaker at 200 rpm. The HRP reacted with the substrate in the presence of H_2_O_2_, producing a visually detectable pink color. After the incubation period, the color change was measured photometrically at a wavelength of 560 nm. The mean values were calculated from all replicates.

### Determination of IMA concentration using a human ischemia modified albumin (IMA) ELISA kit

The Human Ischemia Modified Albumin (IMA) ELISA Kit was purchased from ELISAGenie, UK (# HUEB0871). All reagents from the kit were brought to room temperature (15–25 °C) before the assay. Patient and control sera, previously stored at -20 °C or -80 °C, were thawed at 4 °C. Test strips were inserted into the provided ELISA plate frame, and a protocol sheet was prepared. The first and second rows were designated for the creation of a standard curve (positive and negative controls) and quality assurance, while the remaining rows were used for sample analysis. Serum samples, previously frozen at -20 °C and -80 °C, were thawed to room temperature. Fresh wash buffer was prepared by diluting 30 ml of wash buffer concentrate (25x) with 720 ml deionized water (ddH2O) for each assay. The ELISA washer was programmed and filled with the prepared wash buffer. The standard was diluted with 1,000 µl of sample diluent and resuspended thoroughly for 15 min before use. Detection Reagent A was diluted 1:100 with Assay Diluent A, and Detection Reagent B was diluted 1:100 with Assay Diluent B, according to the required volume. For sample preparation, 96 µl of sample diluent and 24 µl of the serum sample were pipetted into each well, resuspended, and processed in single measurements. A standard curve was prepared: for Standard 1, 300 µl of undiluted standard was pipetted. For Standard 2, 500 µl of undiluted standard was mixed with 500 µl of sample diluent, yielding a 1:2 dilution and a concentration of 100 ng/ml. Subsequent dilutions were prepared by mixing 500 µl of each standard with 500 µl of sample diluent, producing Standards 3 (50 ng/ml), 4 (25 ng/ml), 5 (12.5 ng/ml), 6 (6.25 ng/ml), and 7 (3.125 ng/ml). For the blank, 500 µl of sample diluent was added to the final well. Next, 100 µl of standard, sample, or control was pipetted into the coated wells of the microtiter plate. The plate was gently shaken manually to ensure proper mixing. It was then sealed with adhesive film and incubated in the dark at 37 °C for two hours, due to light sensitivity. After incubation, the liquid was discarded, and 100 µl of freshly prepared Detection Reagent A was added to each well. The plate was sealed again and incubated in the dark at 37 °C for one hour. The wells were washed three times with 400 µl of wash buffer using the ELISA washer, and the excess liquid was removed by tapping the plate on absorbent paper. For the next incubation, 100 µl of Detection Reagent B was added to each well. The plate was gently mixed and incubated again for one hour at 37 °C in the dark, covered with adhesive film. The washing step was repeated five times. To induce the enzyme–substrate chromogen reaction, 90 µl of substrate solution was added to each well and incubated for 10–20 min at 37 °C, protected from light. After the incubation period, or once the color change was visually observed, the reaction was stopped by adding 50 µl of stop solution to each well, causing a color shift in the samples. The color intensity was immediately measured photometrically at a wavelength of 450 nm.

### Ethics

This study was approved by the Ethics Committee of the Brandenburg Medical School (MHB) (No. E-01–20201106). Prior to inclusion, all participants received a detailed explanation and provided written informed consent. All procedures performed in our studies were in accordance with the standards of the institutional and/or national research committee and with the 1964 Helsinki declaration and its later amendments or comparable ethical standards.

### Statistics

Descriptive statistics: Data are presented as mean for normally distributed data, as median for non-parametric data, and as the count or percentage for binary data. Continuous data was tested for normality of distribution by Shapiro–Wilk test.

Inferential statistics: Differences between binary data groups were assessed by chi-square (Χ^2^) test. For non-normal distributed continuous data, differences were assessed by Mann–Whitney U test, or Jonckheere-Terpstra test. The influence of multiple independent parameters on a dependent variable was analyzed in a multivariable logistic regression model. A *p* value < 0.05 was considered significant.

Figures: Data is presented as boxplots, denoting median, first and third quartile, and whiskers at ± 1.5 × interquartile range; outliers are represented as circles.

## Results

From March 2020 until May 2021, 404 individuals where enrolled. These consisted of 166 COVID-19 patients, and 238 controls. Median age in the COVID-19 group was 77 years; 60% were male, 40% were female. Median age in the control group was 39 years; 28% were male, 72% were female. Within the COVID-19 group, 68 patients fell in the clinically”light” subgroup, 57 in the “moderate” subgroup, 19 in the “severe” subgroup and 22 in the “lethal” subgroup (Table 1).

**Table 1:** Age, sex, body mass index, and percentage of patients with more than three comorbidities in the COVID-19 group

Clinical course	Age (years, median)	Sex (male: female)	BMI (Kg/m^2^, median)	> 3 comorbidities (%)
Light (n = 68)	77	59: 41	26	12
Moderate (n = 57)	77	58: 42	29	18
Severe (n = 19)	66	63: 37	30	5
Lethal (n = 22)	83	68: 32	27	0
Significance	p = 0.19	p = 0.84	p = 0.20	p = 0.25

BMI = body mass index

Risk factors for a severe course were frequently present in the COVID-19 group: 68% had hypertension, 35% had type-2 diabetes, and 29% had COPD, nicotine abuse, or other respiratory diseases (Table 2). These comorbidities were mainly treated according to national or international guidelines. However, there was no significant difference regarding the number of comorbidities, age, sex, or body mass index (BMI) between the four subgroups (Table 1).

**Table 2:** Relevant comorbidities in COVID-19 patients

Diagnosis	Patients (%)
Hypertension	68
Diabetes mellitus type 2	35
Coronary disease	27
Chronic heart failure	19
Immunosupression	15
Other respiratory diseases	14
COPD	8
Nicotine abuse	7
History of stroke	5

### Xanthine oxidase

**Fig. 2:**
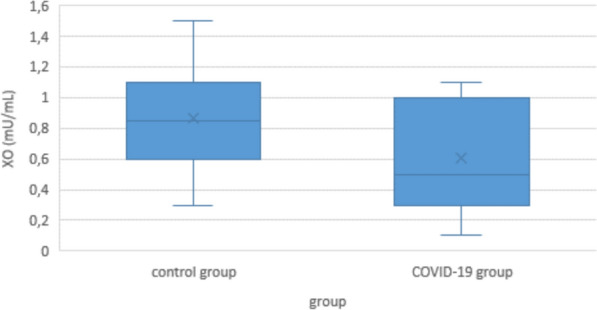
Serum xanthine oxidase (XO) levels of COVID-19 patients and controls

Minimum XO concentration in COVID-19 patients was 0.1 mU/mL, maximum was 1.1 mU/mL; median was 0.5 mU/mL. XO concentration in controls was significantly higher with a median of 0.85 mU/mL (min 0.3 mU/mL; max 1.5 mU/mL; p < 0.001) (Fig. 2).

**Fig. 3:**
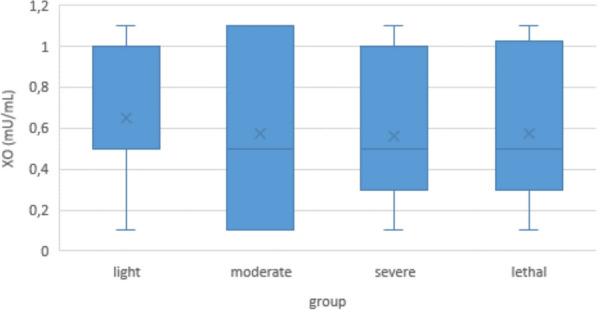
Serum XO levels in COVID-19 patients according to disease severity

Within the COVID-19 group, there were no significant differences in XO between the light, moderate, severe, or lethal subgroups (Fig. 3).

### Superoxide dismutase

**Fig. 4:**
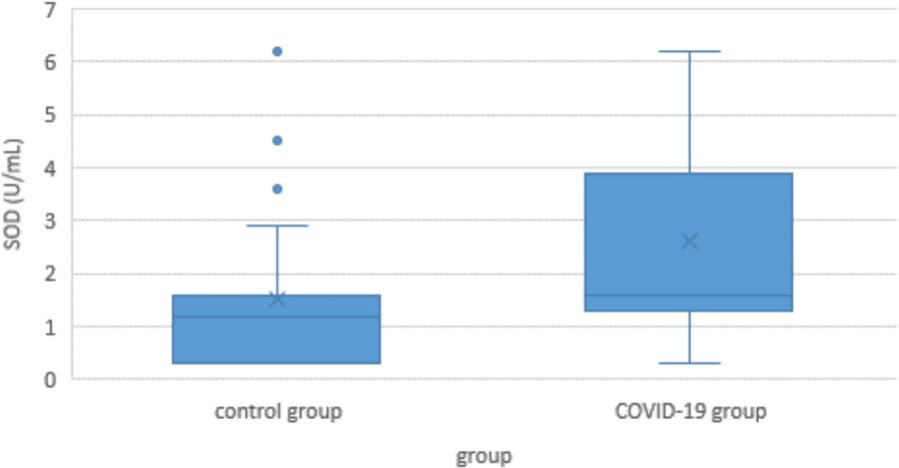
Serum superoxide dismutase (SOD) levels in COVID-19 patients and controls

Minimum SOD concentration in COVID-19 patients was 0.3 U/mL, maximum was 6.2 mU/mL; median was 1.6 U/mL. In the control group, the SOD content showed a similar maximum and minimum of 0.3 and 6.2 U/mL respectively, but because of a differently skewed distribution, the median of 1.2 U/mL was significantly lower (p < 0.001) (Fig. 4).

**Fig. 5:**
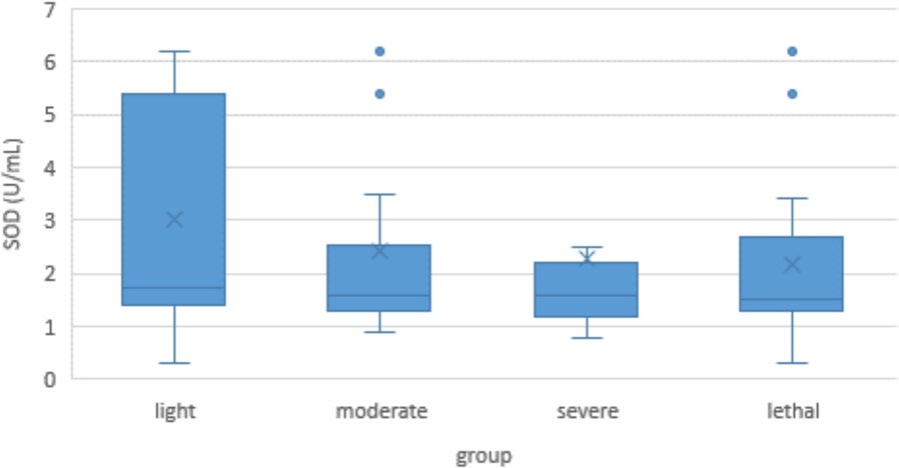
Serum SOD levels in COVID-19 patients according to disease severity

Within the COVID-19 group, there were no significant differences in SOD between the light, moderate, severe or lethal subgroups (Fig. 5).

### Catalase

**Fig. 6:**
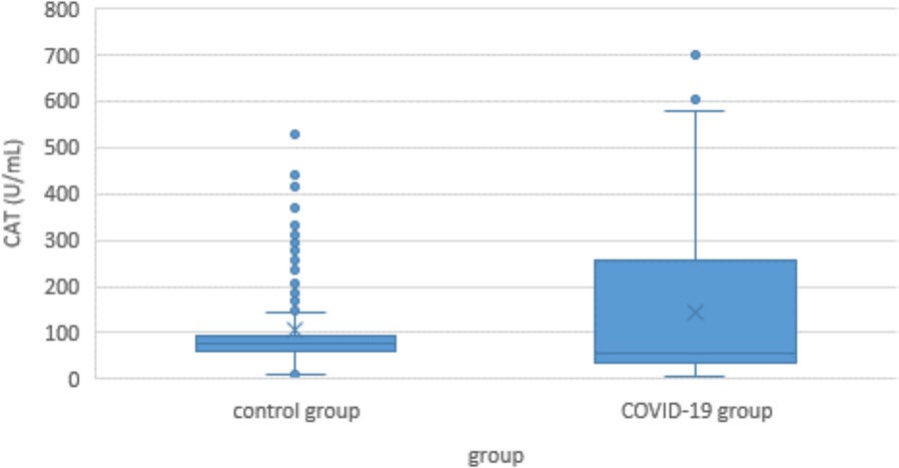
Serum catalase (CAT) levels of COVID-19 patients controls

Minimum CAT concentration in COVID-19 patients was 4.5 U/mL, maximum was 697.6 U/mL; median was 58.0 U/mL. CAT concentration in controls was significantly higher with a median of 76 U/mL (min 8.4 U/mL; max 528.8 U/mL; p < 0.001) (Fig. 6).

**Fig. 7:**
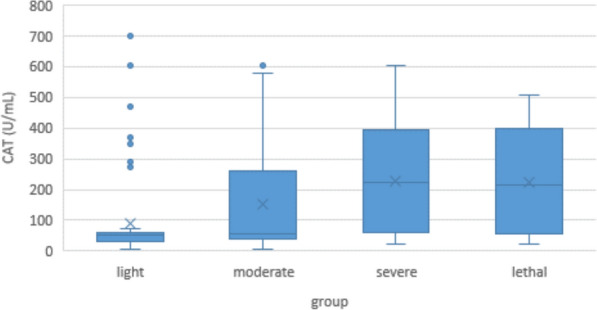
Serum CAT levels in COVID-19 patients according to disease severity

Within the COVID-19 group, CAT concentration was significantly correlated to clinical severity, with 53.4 U/mL in the “light” group, 57.3 U/mL in the “moderate” group, 225.1 U/mL in the “severe” group, and 216.8 U/mL in the ‘lethal’” group. (p < 0.001) (Fig. 7).

### Ischemia-modified albumin

**Fig. 8:**
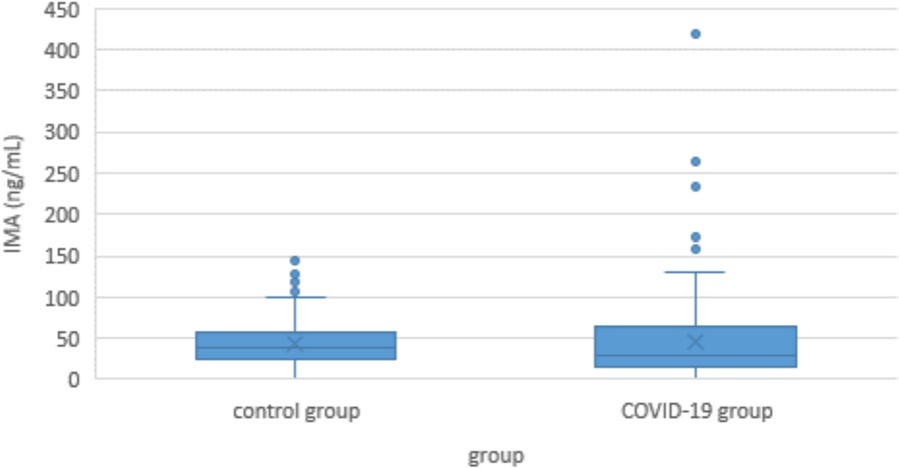
Serum ischemia modified albumin (IMA) levels of COVID-19 patients and controls

**Fig. 9:**
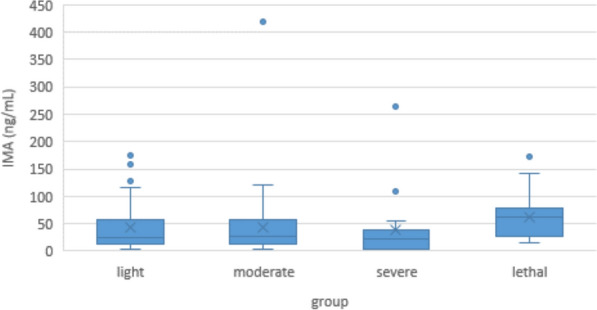
Serum IMA levels in COVID-19 patients according to disease severity

Minimum IMA concentration in COVID-19 patients was 1.8 ng/mL, maximum was 418.7 ng/mL, median was 29,8 ng/mL. In the control group, values were lower but this was not significant. (Minimum 0.5 ng/mL, maximum 145.3 ng/mL, median 37.7 ng/mL; p = 0.1) (Fig. 8). Within the COVID-19 group, there were no significant differences in IMA between the light, moderate, severe or lethal subgroups (Fig. 9).

### Logistic regression analysis

To further investigate the influence of the each predictor on the severity of COVID-19 disease, we performed a multivariate ordinal logistic regression analysis.

In this analysis, only CAT levels showed a significant positive correlation to the severity of the disease. Specifically, if the CAT enzyme activity increased by 1 U/mL, then the probability of being in the next more severe COVID-19 category increased by 0.4%. SOD and XO showed a significant negative correlation: If SOD increased by one unit (1 U/mL), the probability of being in the next more severe subgroup decreased by 20%; if XO increased by one unit (1 mU/mL), the probability of being in the next more severe subgroup decreased by 9.4% (Table 3).

**Table 3:** Ordinal log regression analysis

	Estimate	Std Err	Wald	df	Sig	95% conf
SOD (U/mL)	-0,215	0,092	5,483	1	0,019	-0,395–0,035
CAT (U/mL)	0,004	0,001	13,283	1	< 0,001	0,002–0,006
XO (mU/mL)	-0,94	0,470	3,999	1	0,046	-1,862- -0,019
IMA (ng/mL)	-0,001	0,003	0,071	1	0,79	-0,007 -0,006
BMI (kg/m^2^)	0,057	0,029	3,747	1	0,053	-0,001 -0,114
Age (years)	0,029	0,015	3,557	1	0,059	-0,001–0,058
Sex	-0,207	0,353	0,345	1	0,557	-0,899–0,484
> 3 Comorbidities	-0,332	0,341	0,947	1	0,331	-1,001–0,337

Target variable: clinical course. Std. Err = standard error, df = degrees of freedom, Sig = significance value, 95% conf = 95% confidence interval, CAT = catalase, SOD = superoxide dismutase, XO = xanthine oxidase, IMA = ischemic modified albumin, BMI = body-mass-index

There was no significant correlation of IMA, age, sex, BMI, or number of comorbidities with the severity of the clinical course.

## Discussion

The aim of this study was two-fold. First, we wanted to shed light onto the patho-mechanisms of COVID-19 regarding vascular oxidative stress and oxidant / antioxidant status. Secondly, we expected the results of our oxidant and antioxidant measurements to correlate with the severity of the disease, so that they could be used as potential markers for the overall clinical outcome.

During the study, two main methodological problems arose: The first was the marked age imbalance between the COVID-19 group and the control group (Table 1). One reason for this is that all data was acquired during the height of the pandemic, with all contact restrictions and hygienic regulations in effect. So getting any samples at all was difficult, especially from healthy controls. Second problem was the only partly standardized timing of blood sampling in individual patients. These effects can severely limit interpretability and should be kept im mind when discussing the results (see `Limitations` section below).

### Mechanisms of oxidative stress

#### Xanthine oxidase

It is long known that the concentration of XO in the vascular endothelium is up to 100-fold higher than in other cell types [40]. For every uric acid molecule it produces, two molecules of O_2_^•−^ are generated as well, so it is one mayor source of vascular oxidative stress [49]. What is also known, is that XO activity is increased in various pulmonary viral infections, and it has been hypothesized that this may also play a role in COVID-19 [50–52]. However, indirect investigations on uric acid do not clearly support that hypothesis. While some authors indeed found a positive correlation of disease severity with serum UA [53, 54], others found a negative correlation [55], a U-shaped correlation [56], or none at all [57].

In our own direct measurements of serum XO, we found a slight but significant decrease in COVID-19 patients compared to controls. Correlation with disease severity was ambiguous, with a weak negative correlation in the multivariate analysis, and no significant correlation in the Jonckheere-Terpstra test. (This may be due to the fact that some patients had to be omitted from the multivariate analysis because of missing data, see below.) Even with the inconsistencies between the groups, this might suggest that the leaking of XO from the endothelial intracellular space into the blood stream does not play a crucial role in COVID-19 biology (Figs. 2 and 3). It would also mean that oxidative stress in COVID-19 derives from other sources than endothelial XO, possibly neutrophilic myeloperoxidase, or NADPH oxidase [58–61]. And it would give a possible explanation why the theoretical advantages of allopurinol or febuxostat as a therapy for COVID-19 disease could not be confirmed in clinical studies [62–65].

#### Superoxide dismutase

SOD is long known to be one of the main protective molecules against oxidative stress [42, 66]. In COVID-19, its role is as controversial as XO’s. Some authors found it to be increased in comparison to controls, and to be positively correlated with disease severity [67]. Others reported it to be decreased, and to be negatively correlated [68, 69]. Consistent with that, our own findings are inconsistent: On the one hand, serum concentration of SOD in COVID-19 patients was higher than in controls. On the other hand, correlation of SOD concentration with clinical severeness was negative in the multivariate analysis, and non-significant in the Jonckheere-Terpstra test (Figs. 4 and 5).

Taking these inconsistencies into account, one possible explanation would be that as an enzyme, SOD is not altered by the reaction it catalyzes [43]. So unlike radical scavengers like vitamin C or glutathione that get “used up” by the ROS they eliminate [70], SOD would rather be upregulated by the cytokine storm [71, 72]. Consequently, it is elevated in the COVID-19 group. As the disease progresses, this mechanism might become exhausted, which would result in a secondary decrease of plasma levels with increasing clinical severity. Additionally, these effects may be superimposed by a high inter-individual variance of SOD levels as seen in the control group.

#### Catalase

The role of catalase in COVID-19 is even more controversial than the one of XO or SOD. On the one hand, it is a typical protective enzyme against oxidative stress, so it should be beneficial in any kind of hyperinflammation [46]. On the other hand, gene expression of CAT and ACE2 is positively correlated in the lung, and there is evidence that these molecules interact with each other on the cell surface [73]. So COVID-19 may be the exception of the rule, with high CAT levels being a risk factor rather than a protection. And indeed, some researchers found CAT levels elevated in COVID-19 patients [69]. However, others found them unchanged [74], or to be reduced and negatively correlated to disease severity [75].

Unexpectedly, the latter group found H_2_O_2_ levels substantially reduced too, which could not be explained at that time. Even if our our own results on SOD may be skewed by the inconsistencies between groups (see below), one might speculate that the reduction of SOD with worsening inflammation would lead to a block in the reaction from superoxide anion to hydrogen peroxide, resulting not only in endothelial damage by O2^•−^, but also in a reduced levels of H_2_O_2_. The lower activity of CAT would then be the result of a simple downregulation, or inverse enzyme induction [76].

In our own study, CAT levels were reduced in COVID-19 patients compared to controls, which is in concordance with the above hypothesis. In the subgroups, however, it had a strong positive correlation with clinical severity that is in concordance with other authors but cannot be explained at the time [69] (Figs. 6 and 7).

#### Ischemia-altered albumin

While the mechanisms that lead to oxidative damage in COVID-19 are poorly understood, the fact that this damage exists and that it plays a central role in the proceeding of the disease is undisputed. Accordingly, it should be expected that a general marker for oxidative damage like IMA would be clearly elevated in patients and correlated with disease severity. But this is not the case. While some authors have found a correlation of IMA levels with clinical course or mortality [76, 77], others found no correlation and judged it not helpful in predicting the severity of the disease [78–80]. Our own findings support the latter, although one must concede that the non-standardized timing of blood sampling might again play a role here (Figs. 8 and 9). In all studies, including our own, blood samples were drawn at only one time point, and these time points varied from time of the first positive corona test, time of first CT scan, and time of admission to the hospital, to time of admission to the intensive care unit. Given the half-life of albumin, and the high variance in the XO, SOD, and CAT levels we have seen, this may explain the inconsistency of those results.

### Markers to predict disease severity

Age, obesity, hypertension and concomitant pulmonary diseases are known risk factors in COVID-19. However, these categories are too general to predict the outcome on an individual level, so on-admission laboratory tests to forecast the clinical course are needed. Since early in the pandemic, numerous biomarkers were tried for this purpose, most of them being markers of inflammation like ferritin, CRP or interleukins, markers of organ function, or markers of organ damage [81].

Markers for oxidative stress were rarely tried, maybe because of the technical difficulties in measuring ROS or their oxidation products in vivo [34, 35]. From the few authors who worked on this subject, we know that the outcome is strongly correlated to total oxidant or antioxidant power [67, 82], and that markers like oxidized LDL, or thiol are highly predictive in a clinical setting [78, 83]. So the principle of oxidative stress markers as predictors of disease severity in COVID-19 has been proven.

In our own multivariate analysis, we a found a strong correlation of CAT levels with clinical severity, and weaker correlations for SOD and XO. Since our results may be skewed by the limitations discussed above and below, we propose serum CAT to be further explored in a more closely controlled study as a clinical marker in COVID-19.

### Limitations

This study is not without flaws. First and foremost, differences in age and sex between the COVID-19 group and the control group are considerable confounders when comparing these groups (Table 1). Working with the data we had, and keeping this flaw in mind, we still think that our results are worth reporting. However, we did not incorporate the healthy control group in the multivariate analysis because of this incompatibility (Table 3).

The fact that we could not collect BMI data for all patients was also due to the particular circumstances during the pandemic. Since we had to exclude those patients from the multivariate analysis, this would explain the inconsistency between the analysis and the Jonckheere-Terpstra test. However, between the COVID-19 groups, BMI did not differ significantly, so it is unlikely that the results were substantially skewed by it (Table 1).

Apart from obesity, there are numerous other factors that may have influenced our results. COPD, hypertension, diabetes mellitus type 2, coronary disease, chronic heart failure are typical comorbidities known to increase the likelihood of a severe course in COVID-19 disease. Additionally, the medication needed for some of these conditions, like statins, can influence not only the severeness of the disease but the oxidant / antioxidant status of a patient. And indeed, many of these conditions were present in many of our patients (Table 2). However, when comparing the individual severeness groups, number of comorbidities and severeness were not related. In contrast, there were even more relevant comorbidities in the lighter groups, but with a *p* level of 0.25, these differences were far from significant (Table 1). We still cannot rule out that these factors in some way influenced our results.

Another limitation is that we defined disease severity using a simplified Clinical Progression Scale. This scale differentiates only by care level and respiratory support, but not by objective respiratory or radiological parameters [48]. It could theoretically have lead to a situation were a patient with severe hypoxia was triaged to be left on the normal ward without mechanical ventilation and thus falsely put in the wrong group in our study. However, during the whole course of the pandemic, our center’s intensive care capacity was never exhausted and patients were generally cared for according to non-pandemic standards. We therefore think that such a scenario is rather unlikely but of course we cannot rule it out completely. So while not ideal, the parameters we used seemed best suited to define clinical severeness.

Finally, blood sampling was not standardized relative to symptom onset and was taken at different clinical stages. We did this because we tried to hit the “turning points” of the disease, i.e. admission to the highest standard of medical care. Therefore, we drew samples on the day of admission to the hospital, or on admission to the ICU. Due to logistic difficulties during the pandemic, the time point on admission day was anywhere during that day. The samples from the ICU, on the other hand, were usually drawn within one hour of admission. Other authors working on the subject have faced the same difficulties: MEHRI et al. drew their samples within a time frame of 24 h after admission [67], UYSAL et al. give no information of the time point other than it was in the morning after a fasting period [83], and TEPEBAŞI et al. drew theirs at an unspecified time on admission day [78]. Given the dynamic nature of oxidative stress, this could have contributed to the relatively strong statistical spread within the groups.

To overcome these significant limitations, future investigations could use a longitudinal design with samples drawn depending on objective parameters like blood oxygen saturation, or at pre-defined time points during the course of the disease.

## Data Availability

All relevant data are within the paper.

## Abbreviations

ACE: Angiotensin converting enzyme
BMI: Body mass index
CAT: Catalase
COPD: Chronic obstructive pulmonary disease
COVID-19: Coronavirus 2019
CPS: Clinical progression scale
ddH_2_O: Deionized water
DSMO: Dimethyl sulfoxide
ELISA: Enzyme linked immunoassay
HRP: Horseradish peroxidase
H_2_O_2_: Hydrogen peroxide
IMA: Ischemia modified albumin
NADPH: Nicotinamide adenine dinucleotide phosphate
O_2_•-: Superoxide anion, hyperoxide
ROS: Reactive oxygen species
SOD: Superoxide dismutase
XO: Xanthine oxidase

## References

[1] Agarwal KM, Mohapatra S, Sharma P, Sharma S, Bhatia D, Mishra A. Study and overview of the novel corona virus disease (COVID-19). Sens Int. 2020;1:100037. 10.1016/j.sintl.2020.100037. 34766042 10.1016/j.sintl.2020.100037 PMC7474965

[2] Gupta A, Madhavan MV, Sehgal K, et al. Extrapulmonary manifestations of COVID-19. Nat Med. 2020;26(7):1017–32. 10.1038/s41591-020-0968-3. 32651579 10.1038/s41591-020-0968-3 PMC11972613

[3] Spike-ACE2 protein-protein interaction (AlphaLISA). In: SARS-CoV-2 Assays. Bethesda (MD): National Center for Advancing Translational Sciences (NCATS); 2020. 35512044

[4] Bourgonje AR, Abdulle AE, Timens W, et al. Angiotensin-converting enzyme 2 (ACE2), SARS-CoV-2 and the pathophysiology of coronavirus disease 2019 (COVID-19). J Pathol. 2020;251(3):228–48. 10.1002/path.5471. 32418199 10.1002/path.5471 PMC7276767

[5] Tsampasian V, Bäck M, Bernardi M, Cavarretta E, Dębski M, Gati S, et al. Cardiovascular disease as part of long COVID: a systematic review. Eur J Prev Cardiol. 2024. 10.1093/eurjpc/zwae070. 10.1093/eurjpc/zwae070 38381595

[6] Bernard I, Limonta D, Mahal LK, Hobman TC. Endothelium infection and dysregulation by SARS-CoV-2: evidence and caveats in COVID-19. Viruses. 2020;13(1):29. 10.3390/v13010029. 33375371 10.3390/v13010029 PMC7823949

[7] Cheon SY, Koo BN. Inflammatory response in COVID-19 patients resulting from the interaction of the inflammasome and SARS-CoV-2. Int J Mol Sci. 2021;22(15):7914. 10.3390/ijms22157914. 34360684 10.3390/ijms22157914 PMC8348456

[8] Mehta P, McAuley DF, Brown M, et al. COVID-19: consider cytokine storm syndromes and immunosuppression. Lancet. 2020;395(10229):1033–4. 10.1016/S0140-6736(20)30628-0. 32192578 10.1016/S0140-6736(20)30628-0 PMC7270045

[9] Birnhuber A, Fließer E, Gorkiewicz G, et al. Between inflammation and thrombosis: endothelial cells in COVID-19. Eur Respir J. 2021;58(3):2100377. 10.1183/13993003.00377-2021. 33958433 10.1183/13993003.00377-2021 PMC8112008

[10] Conway EM, Mackman N, Warren RQ, et al. Understanding COVID-19-associated coagulopathy. Nat Rev Immunol. 2022;22(10):639–49. 10.1038/s41577-022-00762-9. 35931818 10.1038/s41577-022-00762-9 PMC9362465

[11] Chernyak BV, Popova EN, Prikhodko AS, Grebenchikov OA, Zinovkina LA, Zinovkin RA. COVID-19 and oxidative stress. Biochemistry (Mosc). 2020;85(12):1543–53. 10.1134/S0006297920120068. 33705292 10.1134/S0006297920120068 PMC7768996

[12] Paul O, Akolia IK, Qin Tao J, et al. Reactive oxygen species in endothelial signaling in COVID-19: protective role of the novel peptide PIP-2. PLoS ONE. 2024;19(5):e0289854. 10.1371/journal.pone.0289854. 38771750 10.1371/journal.pone.0289854 PMC11108150

[13] Cooke JP, Connor JH, Jain A. Acute and chronic cardiovascular manifestations of COVID-19: role for endotheliopathy. Methodist Debakey Cardiovasc J. 2021;17(5):53–62. 10.14797/mdcvj.1044. 34992723 10.14797/mdcvj.1044 PMC8680072

[14] Witting PK, Rayner BS, Wu BJ, Ellis NA, Stocker R. Hydrogen peroxide promotes endothelial dysfunction by stimulating multiple sources of superoxide anion radical production and decreasing nitric oxide bioavailability. Cell Physiol Biochem. 2007;20(5):255–68. 10.1159/000107512. 17762155 10.1159/000107512

[15] Jankauskas SS, Kansakar U, Sardu C, et al. COVID-19 causes ferroptosis and oxidative stress in human endothelial cells. Antioxidants (Basel). 2023;12(2):326. 10.3390/antiox12020326. 36829885 10.3390/antiox12020326 PMC9952002

[16] Montiel V, Lobysheva I, Gérard L, et al. Oxidative stress-induced endothelial dysfunction and decreased vascular nitric oxide in COVID-19 patients. EBioMedicine. 2022;77:103893. 10.1016/j.ebiom.2022.103893. 35219085 10.1016/j.ebiom.2022.103893 PMC8865837

[17] Zhang Q, Huang X. Induction of interleukin-6 by coal containing bioavailable iron is through both hydroxyl radical and ferryl species. J Biosci. 2003;28(1):95–100. 10.1007/BF02970138. 12682431 10.1007/BF02970138

[18] Mroueh A, Fakih W, Carmona A, et al. COVID-19 promotes endothelial dysfunction and thrombogenicity: role of proinflammatory cytokines/SGLT2 prooxidant pathway. J Thromb Haemost. 2024;22(1):286–99. 10.1016/j.jtha.2023.09.022. 37797691 10.1016/j.jtha.2023.09.022

[19] Gencer S, Lacy M, Atzler D, van der Vorst EPC, Döring Y, Weber C. Immunoinflammatory, thrombohaemostatic, and cardiovascular mechanisms in COVID-19. Thromb Haemost. 2020;120(12):1629–41. 10.1055/s-0040-1718735. 33124029 10.1055/s-0040-1718735 PMC7869061

[20] Pisoschi AM, Pop A. The role of antioxidants in the chemistry of oxidative stress: a review. Eur J Med Chem. 2015;97:55–74. 10.1016/j.ejmech.2015.04.040. 25942353 10.1016/j.ejmech.2015.04.040

[21] Soto ME, Guarner-Lans V, Díaz-Díaz E, et al. Hyperglycemia and loss of redox homeostasis in COVID-19 patients. Cells. 2022;11(6):932. 10.3390/cells11060932. 35326383 10.3390/cells11060932 PMC8946177

[22] Rovas A, Osiaevi I, Buscher K, et al. Microvascular dysfunction in COVID-19: the MYSTIC study. Angiogenesis. 2021;24(1):145–57. 10.1007/s10456-020-09753-7. 33058027 10.1007/s10456-020-09753-7 PMC7556767

[23] Silva MJA, Ribeiro LR, Gouveia MIM, et al. Hyperinflammatory response in COVID-19: a systematic review. Viruses. 2023;15(2):553. 10.3390/v15020553. 36851766 10.3390/v15020553 PMC9962879

[24] Siddiqi HK, Libby P, Ridker PM. COVID-19 - a vascular disease. Trends Cardiovasc Med. 2021;31(1):1–5. 10.1016/j.tcm.2020.10.005. 33068723 10.1016/j.tcm.2020.10.005 PMC7556303

[25] Oudkerk M, Kuijpers D, Oudkerk SF, van Beek EJ. The vascular nature of COVID-19. Br J Radiol. 2020;93(1113):20200718. 10.1259/bjr.20200718. 32735457 10.1259/bjr.20200718 PMC7465848

[26] Li M, Zhu D, Yang J, et al. Clinical treatment experience in severe and critical COVID-19. Mediators Inflamm. 2021;2021(1):9924542. 10.1155/2021/9924542. 34602859 10.1155/2021/9924542 PMC8483935

[27] García LF. Immune response, inflammation, and the clinical spectrum of COVID-19. Front Immunol. 2020;11:1441. 10.3389/fimmu.2020.01441. (**Published 2020 Jun 16**). 32612615 10.3389/fimmu.2020.01441 PMC7308593

[28] Gianni P, Goldin M, Ngu S, Zafeiropoulos S, Geropoulos G, Giannis D. Complement-mediated microvascular injury and thrombosis in the pathogenesis of severe COVID-19: a review. World J Exp Med. 2022;12(4):53–67. 10.5493/wjem.v12.i4.53. (**Published 2022 Jul 20**). 36157337 10.5493/wjem.v12.i4.53 PMC9350720

[29] Colantuoni A, Martini R, Caprari P, et al. COVID-19 sepsis and microcirculation dysfunction. Front Physiol. 2020;11:747. 10.3389/fphys.2020.00747. (**Published 2020 Jun 26**). 32676039 10.3389/fphys.2020.00747 PMC7333313

[30] Del Valle DM, Kim-Schulze S, Huang HH, et al. An inflammatory cytokine signature predicts COVID-19 severity and survival. Nat Med. 2020;26(10):1636–43. 10.1038/s41591-020-1051-9. 32839624 10.1038/s41591-020-1051-9 PMC7869028

[31] Townsend L, Dyer AH, Naughton A, et al. Severe COVID-19 is characterised by inflammation and immature myeloid cells early in disease progression. Heliyon. 2022;8(4):e09230. 10.1016/j.heliyon.2022.e09230. 35386227 10.1016/j.heliyon.2022.e09230 PMC8973020

[32] Majumder N, Deepak V, Hadique S, et al. Redox imbalance in COVID-19 pathophysiology. Redox Biol. 2022;56:102465. 10.1016/j.redox.2022.102465. 36116160 10.1016/j.redox.2022.102465 PMC9464257

[33] Murphy MP, Bayir H, Belousov V, et al. Guidelines for measuring reactive oxygen species and oxidative damage in cells and in vivo. Nat Metab. 2022;4(6):651–62. 10.1038/s42255-022-00591-z. 35760871 10.1038/s42255-022-00591-z PMC9711940

[34] Brieger K, Schiavone S, Miller FJ Jr, Krause KH. Reactive oxygen species: from health to disease. Swiss Med Wkly. 2012;142:w13659. 10.4414/smw.2012.13659. (**Published 2012 Aug 17**). 22903797 10.4414/smw.2012.13659

[35] Berkowitz BA. Oxidative stress measured in vivo without an exogenous contrast agent using QUEST MRI. J Magn Reson. 2018;291:94–100. 10.1016/j.jmr.2018.01.013. 29705036 10.1016/j.jmr.2018.01.013 PMC5963509

[36] Ayala JC, Grismaldo A, Sequeda-Castañeda LG, Aristizábal-Pachón AF, Morales L. Oxidative stress in ICU patients: ROS as mortality long-term predictor. Antioxidants Basel. 2021;10(12):1912. 10.3390/antiox10121912. (**Published 2021 Nov 29**). 34943015 10.3390/antiox10121912 PMC8750443

[37] Zamora PL, Villamena FA. Clinical Probes for ROS and Oxidative Stress. In: Berliner LJ, Parinandi NL, eds. Measuring Oxidants and Oxidative Stress in Biological Systems. Cham (CH): Springer; August 9, 2020.13–38. 33411445

[38] Menger KE, Logan A, Luhmann UFO, et al. In vivo measurement of mitochondrial ROS production in mouse models of photoreceptor degeneration. Redox Biochem and Chem. 2023. 10.1016/j.rbc.2023.100007. 10.1016/j.rbc.2023.100007 PMC10686909 38046619

[39] Parks DA, Granger DN. Xanthine oxidase: biochemistry, distribution and physiology. Acta Physiol Scand Suppl. 1986;548:87–99. 3529824

[40] Jarasch ED, Bruder G, Heid HW. Significance of xanthine oxidase in capillary endothelial cells. Acta Physiol Scand Suppl. 1986;548:39–46. 3463124

[41] Jarasch ED, Grund C, Bruder G, Heid HW, Keenan TW, Franke WW. Localization of xanthine oxidase in mammary-gland epithelium and capillary endothelium. Cell. 1981;25(1):67–82. 10.1016/0092-8674(81)90232-4. 6895049 10.1016/0092-8674(81)90232-4

[42] Nozik-Grayck E, Suliman HB, Piantadosi CA. Extracellular superoxide dismutase. Int J Biochem Cell Biol. 2005;37(12):2466–71. 10.1016/j.biocel.2005.06.012. 16087389 10.1016/j.biocel.2005.06.012

[43] He T, Peterson TE, Holmuhamedov EL, et al. Human endothelial progenitor cells tolerate oxidative stress due to intrinsically high expression of manganese superoxide dismutase. Arterioscler Thromb Vasc Biol. 2004;24(11):2021–7. 10.1161/01.ATV.0000142810.27849.8f. 15319267 10.1161/01.ATV.0000142810.27849.8f

[44] Faraci FM, Didion SP. Vascular protection: superoxide dismutase isoforms in the vessel wall. Arterioscler Thromb Vasc Biol. 2004;24(8):1367–73. 10.1161/01.ATV.0000133604.20182.cf. 15166009 10.1161/01.ATV.0000133604.20182.cf

[45] Marklund SL, Westman NG, Lundgren E, Roos G. Copper- and zinc-containing superoxide dismutase, manganese-containing superoxide dismutase, catalase, and glutathione peroxidase in normal and neoplastic human cell lines and normal human tissues. Cancer Res. 1982;42(5):1955–61. 7066906

[46] Shevtsova A, Gordiienko I, Tkachenko V, Ushakova G. Ischemia-modified albumin: origins and clinical implications. Dis Markers. 2021;2021:9945424. 10.1155/2021/9945424. (**Published 2021 Jul 19**). 34336009 10.1155/2021/9945424 PMC8315882

[47] An ordinal severity scale for COVID-19 retrospective studies using Electronic Health Record data. JAMIA Open. 2022;5(3):ooac066. 022 Jul 9. 10.1093/jamiaopen/ooac066 10.1093/jamiaopen/ooac066 PMC9278199 35911666

[48] WHO Working Group on the Clinical Characterisation and Management of COVID-19 infection. A minimal common outcome measure set for COVID-19 clinical research. Lancet Infect Dis. 2020 Aug;20(8):e192-e197. 10.1016/S1473-3099(20)30483-7. Epub 2020 Jun 12. Erratum in: Lancet Infect Dis. 2020 Oct;20(10):e250. doi: 10.1016/S1473-3099(20)30637-X. PMID: 32539990; PMCID: PMC7292605. 10.1016/S1473-3099(20)30483-7 PMC7292605 32539990

[49] Li H, Horke S, Förstermann U. Oxidative stress in vascular disease and its pharmacological prevention. Trends Pharmacol Sci. 2013;34(6):313–9. 10.1016/j.tips.2013.03.007. 23608227 10.1016/j.tips.2013.03.007

[50] Fonseca W, Malinczak CA, Schuler CF, et al. Uric acid pathway activation during respiratory virus infection promotes Th2 immune response via innate cytokine production and ILC2 accumulation. Mucosal Immunol. 2020;13(4):691–701. 10.1038/s41385-020-0264-z. 32047272 10.1038/s41385-020-0264-z PMC7316593

[51] Papi A, Contoli M, Gasparini P, et al. Role of xanthine oxidase activation and reduced glutathione depletion in rhinovirus induction of inflammation in respiratory epithelial cells. J Biol Chem. 2008;283(42):28595–606. 10.1074/jbc.M805766200. 18678861 10.1074/jbc.M805766200 PMC2661410

[52] Pratomo IP, Noor DR, Kusmardi K, et al. Xanthine Oxidase-induced inflammatory responses in respiratory epithelial cells: a review in immunopathology of COVID-19. Int J Inflam. 2021;2021:1653392. 10.1155/2021/1653392. (**Published 2021 Aug 5**). 34367545 10.1155/2021/1653392 PMC8346299

[53] Zheng T, Liu X, Wei Y, et al. Laboratory predictors of COVID-19 mortality: a retrospective analysis from Tongji Hospital in Wuhan. Mediators Inflamm. 2021;2021:6687412. 10.1155/2021/6687412. (**Published 2021 Feb 23**). 33679237 10.1155/2021/6687412 PMC7906000

[54] G S, Balaraj K, Prabhu R, Mukherjee T, Sc R. Can Uric Acid be Used as a Prognostic Factor to Determine the Severity of Covid 19 Infection. J Assoc Physicians India. 2022;70(4):11–12.

[55] Dufour I, Werion A, Belkhir L, et al. Serum uric acid, disease severity and outcomes in COVID-19. Crit Care. 2021;25(1):212. 10.1186/s13054-021-03616-3. (**Published 2021 Jun 14**). 34127048 10.1186/s13054-021-03616-3 PMC8201458

[56] Chen B, Lu C, Gu HQ, et al. Serum uric acid concentrations and risk of adverse outcomes in patients with COVID-19. Front Endocrinol (Lausanne). 2021;12:633767. 10.3389/fendo.2021.633767. (**Published 2021 May 6**). 34025575 10.3389/fendo.2021.633767 PMC8134697

[57] Parmaksız E, Parmaksız ET. Uric acid as a prognostic predictor in COVID-19. Pak J Med Sci. 2022;38(8):2246–52. 10.12669/pjms.38.8.6636. 36415243 10.12669/pjms.38.8.6636 PMC9676607

[58] Barciszewska AM. Elucidating of oxidative distress in COVID-19 and methods of its prevention. Chem Biol Interact. 2021;344:109501. 10.1016/j.cbi.2021.109501. 33974898 10.1016/j.cbi.2021.109501 PMC8106523

[59] Badawy MA, Yasseen BA, El-Messiery RM, et al. Neutrophil-mediated oxidative stress and albumin structural damage predict COVID-19-associated mortality. Elife. 2021;10:e69417. 10.7554/eLife.69417. (**Published 2021 Nov 25**). 34821549 10.7554/eLife.69417 PMC8641949

[60] de Oliveira AA, Nunes KP. Crosstalk of TLR4, vascular NADPH oxidase, and COVID-19 in diabetes: what are the potential implications? Vascul Pharmacol. 2021;139:106879. 10.1016/j.vph.2021.106879. 34051372 10.1016/j.vph.2021.106879 PMC8152239

[61] Goud PT, Bai D, Abu-Soud HM. A multiple-hit hypothesis involving reactive oxygen species and myeloperoxidase explains clinical deterioration and fatality in COVID-19. Int J Biol Sci. 2021;17(1):62–72. 10.7150/ijbs.51811. (**Published 2021 Jan 1**). 33390833 10.7150/ijbs.51811 PMC7757048

[62] Pratomo IP, Ariane A, Tedjo A, Heryanto R, Paramita RI. Xanthine oxidase inhibition in SARS-CoV-2 infection: the mechanism and potency of allopurinol and febuxostat in COVID-19 management. Med J Indones. 2020. 10.13181/mji.rev.204641.

[63] Monserrat Villatoro J, Mejía-Abril G, Díaz García L, et al. A case-control of patients with COVID-19 to explore the association of previous hospitalisation use of medication on the mortality of COVID-19 disease: a propensity score matching analysis. Pharmaceuticals (Basel). 2022;15(1):78. 10.3390/ph15010078. (**Published 2022 Jan 8**). 35056135 10.3390/ph15010078 PMC8780256

[64] Zhang K, Chen R, Jiang Q. Allopurinol increased the risk of COVID-19 hospitalization mediated by E-Selectin downregulation. J Infect. 2023;86(6):620–1. 10.1016/j.jinf.2023.02.030. 36822412 10.1016/j.jinf.2023.02.030 PMC9942479

[65] Davoodi L, Abedi SM, Salehifar E, et al. Febuxostat therapy in outpatients with suspected COVID-19: a clinical trial. Int J Clin Pract. 2020;74(11):e13600. 10.1111/ijcp.13600. 32603531 10.1111/ijcp.13600 PMC7361151

[66] Markey BA, Phan SH, Varani J, Ryan US, Ward PA. Inhibition of cytotoxicity by intracellular superoxide dismutase supplementation. Free Radic Biol Med. 1990;9(4):307–14. 10.1016/0891-5849(90)90005-4. 2126522 10.1016/0891-5849(90)90005-4

[67] Mehri F, Rahbar AH, Ghane ET, Souri B, Esfahani M. Changes in oxidative markers in COVID-19 patients. Arch Med Res. 2021;52(8):843–9. 10.1016/j.arcmed.2021.06.004. 34154831 10.1016/j.arcmed.2021.06.004 PMC8180845

[68] Chu J, Hua L, Liu X, et al. Superoxide dismutase alterations in COVID-19: implications for disease severity and mortality prediction in the context of omicron variant infection. Front Immunol. 2024;15:1362102. 10.3389/fimmu.2024.1362102. (**Published 2024 Feb 22**). 38464514 10.3389/fimmu.2024.1362102 PMC10921560

[69] Kumar DS, Hanumanram G, Suthakaran PK, Mohanan J, Nair LDV, Rajendran K. Extracellular oxidative stress markers in COVID-19 patients with diabetes as co-morbidity. Clin Pract. 2022;12(2):168–76. 10.3390/clinpract12020021. (**Published 2022 Feb 28**). 35314591 10.3390/clinpract12020021 PMC8938798

[70] Pincemail J, Cavalier E, Charlier C, et al. Oxidative stress status in COVID-19 patients hospitalized in intensive care unit for severe pneumonia. A pilot study. Antioxidants (Basel). 2021;10(2):257. 10.3390/antiox10020257. 33562403 10.3390/antiox10020257 PMC7914603

[71] Zuo J, Zhao M, Liu B, et al. TNF-α-mediated upregulation of SOD-2 contributes to cell proliferation and cisplatin resistance in esophageal squamous cell carcinoma. Oncol Rep. 2019;42(4):1497–506. 10.3892/or.2019.7252. 31364751 10.3892/or.2019.7252

[72] Chen X, Choi IY, Chang TS, et al. Pretreatment with interferon-γ protects microglia from oxidative stress via up-regulation of Mn-SOD. Free Radic Biol Med. 2009;46(8):1204–10. 10.1016/j.freeradbiomed.2009.01.027. 19439213 10.1016/j.freeradbiomed.2009.01.027

[73] Qian Y, Li Y, Liu X, et al. Evidence for CAT gene being functionally involved in the susceptibility of COVID-19. FASEB J. 2021;35(4):e21384. 10.1096/fj.202100008. 33710662 10.1096/fj.202100008 PMC8250337

[74] Smail SW, Babaei E, Amin K. Hematological, inflammatory, coagulation, and oxidative/antioxidant biomarkers as predictors for severity and mortality in COVID-19: a prospective cohort-study. Int J Gen Med. 2023;16:565–80. 10.2147/IJGM.S402206. (**Published 2023 Feb 17**). 36824986 10.2147/IJGM.S402206 PMC9942608

[75] Cavalcanti LF, Chagas Silva I, do Nascimento THD, et al. Decreased plasma H2O2 levels are associated with the pathogenesis leading to COVID-19 worsening and mortality. Free Radic Res. 2022;56(11–12):740–8. 10.1080/10715762.2023.2174021. 36708322 10.1080/10715762.2023.2174021

[76] Acar T, Ertekin B, Yortanli M. Value of thiol and ischemia modified albumin (IMA) in predicting mortality in serious COVID-19 pneumonia. Heliyon. 2022;8(12):e12514. 10.1016/j.heliyon.2022.e12514. 36573112 10.1016/j.heliyon.2022.e12514 PMC9771579

[77] Tanrıverdi M, Gündoğdu N, Benlier N, et al. Could ischemia-modified albumin levels predict the severity of disease in SARS-CoV-2 infection? J Infect Dev Ctries. 2023;17(8):1055–62. 10.3855/jidc.17456. (**Published 2023 Aug 31**). 37699088 10.3855/jidc.17456

[78] Tepebaşı MY, İlhan İ, Temel EN, Sancer O, Öztürk Ö. Investigation of inflammation, oxidative stress, and DNA damage in COVID-19 patients. Cell Stress Chaperones. 2023;28(2):191–9. 10.1007/s12192-023-01330-3. 36797451 10.1007/s12192-023-01330-3 PMC9936118

[79] Altintas E, Sabirli R, Yuksekkaya E, Kurt O, Koseler A. Evaluation of serum ischemia modified albumin in patients with COVID-19 pneumonia: a case-control study. Cureus. 2022;14(8):e28334. 10.7759/cureus.28334. (**Published 2022 Aug 24**). 36168388 10.7759/cureus.28334 PMC9500556

[80] Aykac K, Ozsurekci Y, Yayla BCC, et al. Oxidant and antioxidant balance in patients with COVID-19. Pediatr Pulmonol. 2021;56(9):2803–10. 10.1002/ppul.25549. 34265172 10.1002/ppul.25549 PMC8441878

[81] Schmidt W, Jóźwiak B, Czabajska Z, Pawlak-Buś K, Leszczynski P. On-admission laboratory predictors for developing critical COVID-19 during hospitalization - a multivariable logistic regression model. Ann Agric Environ Med. 2022;29(2):274–80. 10.26444/aaem/145376. 35767763 10.26444/aaem/145376

[82] Zendelovska D, Atanasovska E, Petrushevska M, et al. Evaluation of oxidative stress markers in hospitalized patients with moderate and severe COVID-19. Rom J Intern Med. 2021;59(4):375–83. 10.2478/rjim-2021-0014. (**Published 2021 Nov 20**). 33910269 10.2478/rjim-2021-0014

[83] Uysal P, Yüksel A, Durmus S, Cuhadaroglu Ç, Gelisgen R, Uzun H. Can circulating oxidative stress-related biomarkers be used as an early prognostic marker for COVID-19? Front Med. 2023;10:1041115. 10.3389/fmed.2023.1041115. (**Published 2023 Feb 9**). 10.3389/fmed.2023.1041115 PMC9948026 36844214

